# Efficacy of Zhibitai combined with Atorvastatin on blood lipid and inflammation in patients with coronary heart disease

**DOI:** 10.12669/pjms.41.8.10502

**Published:** 2025-08

**Authors:** Shixin Kang, Hongmei Zhao, Nuan Xiao, Linya Zhao, Liangliang Bi

**Affiliations:** 1 Shixin Kang, Department of Cardiovascular Medicine, Affiliated Hospital of Hebei University, Baoding 071000, Hebei, China; 2 Hongmei Zhao, Department of Geriatrics, Affiliated Hospital of Hebei University, Baoding 071000, Hebei, China; 3 Nuan Xiao, Department of Cardiovascular Medicine, Affiliated Hospital of Hebei University, Baoding 071000, Hebei, China; 4 Linya Zhao, Department of Cardiovascular Medicine, Affiliated Hospital of Hebei University, Baoding 071000, Hebei, China; 5 Liangliang Bi, Department of Ultrasonography, Affiliated Hospital of Hebei University, Baoding 071000, Hebei, China

**Keywords:** Atorvastatin, Blood lipids, Coronary heart disease, Effects, Inflammation, Zhibitai capsule

## Abstract

**Objective:**

To evaluate the efficacy and safety of Zhibitai combined with atorvastatin on blood lipid and inflammation.

**Method:**

This was a retrospective study which included eighty-five patients with coronary heart disease (CHD) with dyslipidemia in the Affiliated Hospital of Hebei University, from July 2022 to January 2024. They were divided into two groups according to the random number table, the atorvastatin monotherapy group (n=37) and the combination treatment group (n=48). Observation of changes in blood lipids, inflammatory mediators and incidence of adverse reactions were noted.

**Results:**

The levels of TC, LDL-C, and TG in both groups were significantly reduced compared to pre-treatment (p<0.05. The combination treatment group showed more significant decrease than the monotherapy group at four and 12 weeks of treatment (p<0.05); Both groups showed significant increase in HDL-C levels compared to pre-treatment (p<0.05), at week 12 of treatment, the combination treatment group showed more significant increase compared to the monotherapy group (p<0.05); Both groups can significantly reduce hs-CRP and IL-6(p<0.05); IL-6 was significantly reduced in the combination treatment group at four and 12 weeks of treatment compared to the monotherapy group (p<0.05); The decrease in hs-CRP in the combination treatment group was more significant than the monotherapy group after 12 weeks of treatment (p<0.05). There was no statistically significant difference in the incidence of adverse reactions between the two groups (p>0.05).

**Conclusion:**

Zhibitai combined with atorvastatin can effectively improve the lipid composition of patients with CHD, reduce inflammatory reactions, and have good safety.

## INTRODUCTION

Coronary heart disease(CHD) is one of the common clinical disease,^1^ it mainly affects the blood supply of the heart due to vasospasm, stenosis, blockage, etc. caused by atherosclerotic plaque, resulting in myocardial ischemia and hypoxia injury or even necrosis, threatening the life safety of patients. Hyperlipidemia refers to a pathological increase in blood lipid levels,^2^ studies have shown that hyperlipidemia is a risk factor for the development of coronary heart disease.^3^ Coronary heart disease and hyperlipidemia often coexist and can affect each other, worsen the condition, and lead to poor prognosis for patients. Timely treatment should be initiated in clinical practice, which has a positive impact on improving patient prognosis. Moreover, lipid-lowering is the key to the treatment of coronary heart disease combined with hyperlipidemia. Atorvastatin and other statins are commonly used drugs for regulating blood lipids, however, some patients cannot control their blood lipids within the ideal range simply by using this drug. Long term use of atorvastatin for lipid-lowering may result in adverse reactions, and the overall effect cannot meet clinical expectations.^4^ Zhibitai Capsules are traditional Chinese medicine lipid-lowering preparations that have the effects of unblocking meridians, relieving pain, nourishing qi, and promoting blood circulation, and can also have a lipid-lowering effect.^5^ Based on this, this study aimed to compare the efficacy and safety of Zhibitai capsules in combination with atorvastatin and atorvastatin alone in patients with dyslipidemia, providing the most direct evidence for the combination of Zhibitai capsules and statins.

## METHODS

This was a retrospective analysis study which included 85(49 males and 36 females, aged 50-78 years old) newly diagnosed CHD who were admitted to the Affiliated Hospital of Hebei University between July 2022 to January 2024. They were divided into two groups according to the random number table, namely the monotherapy group (atorvastatin 20 mg QN, n=37) and the combination treatment group (atorvastatin 20 mg QN + Zhibitai capsule 0.48g Bid, n=48). All subjects enrolled in the study met the inclusion and exclusion criteria.

### Ethical Approval:

The study was approved by the Institutional Ethics Committee of Affiliated Hospital of Hebei University (No.: HDFY-LL-2021-067; date: March 11, 2021), and written informed consent was obtained from all participants.

### Inclusion criteria:

- Patients were diagnosed as CHD with symptoms of angina pectoris, coronary CTA suggesting that the degree of stenosis of one or more vessels was ≥50%, and no stents planted;
- Met the inclusion criteria of CHD and were complicated with hyperlipidemia, which, according to the Chinese Guidelines for the Prevention and Treatment of Adult Dyslipidemia (2016 revised edition);
- Met with one or more of the followings: TC≥5.17 mmol/L and/or LDL≥3.1 mmol/L; 2.3≤TG≤5.7 mmol/L; HDL≤1.04 mmol/L (for males) or ≤1.17 mmol/L (for females);
- All patients enrolled were untreated or with irregular lipid-lowering treatment.

### Exclusion criteria:

- Patients with past allergy to atorvastatin and/or Zhibitai, rhabdomyolysis, myositis, severe liver damage (transaminase≥3x upper limits of normal [ULN]);
- Acute myocardial infarction within three months, cerebrovascular disease and heart failure (NYHA III or IV, LVEF <40%) within six months;
- Nephrotic syndrome, hypothyroidism, acute or chronic liver and gallbladder disease, familial hypercholesterolemia, and drug-induced secondary hyperlipidemia.

Both groups received treatment immediately after admission, patients in the two groups were treated with optimized therapeutic regimes according to the guidelines for coronary heart disease treatment issued by the Chinese Health and Family Planning Commission in 2016, i.e., antiplatelet drugs: aspirin 100 mg qd; and patients with hypertension were given amlodipine besylate 5mg qd or bid (to control blood pressure to less than 140/90mmHg). Patients in the monotherapy group were treated with atorvastatin 20 mg QN for 12 weeks and those in the combination treatment group with atorvastatin 20 mg QN combined with Zhibitai capsule 0.48 g Bid for 12 weeks. Atorvastatin was manufactured by Beijing Jialin Pharmaceutical Co., Ltd. (License no. H19990258) and Zhibitai capsule by Chengdu Diao Jiuhong Pharmaceutical Factory (License no. Z51022196).

### Observation indicators:

Collected fasting venous blood after eight hours pre-treatment and at 4-12 weeks of treatment, and measured TC, LDL-C, HDL-C, TG, Liver function, creatine kinase (CK), hsCRP, and IL-6 levels. We also observed the occurrence of adverse reactions in two groups of patients, including gastrointestinal reactions, elevated liver transaminase, muscle pain, etc.

### Statistical analysis:

SPSS25.0 statistical software was used to process the data. Experimental data were presented as mean ± standard deviation ( x±S). Independent t-test was used to compare mean values between two samples and repeated measurement analysis of variance was used to compare the mean values between groups. P<0.05 was considered statistically significant.

## RESULTS

**Fig.1:**
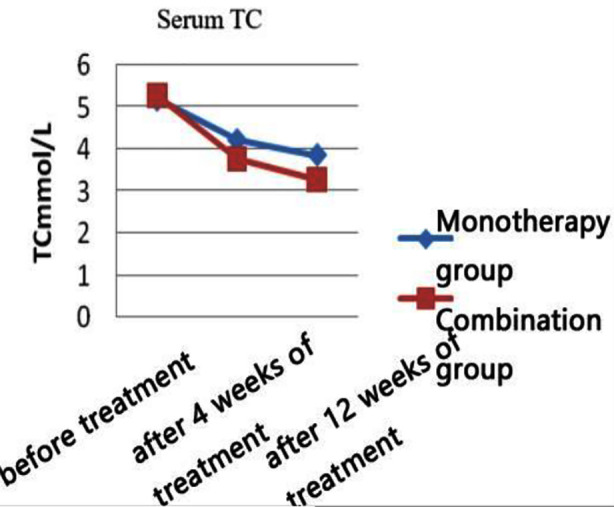
TC levels at each time point in the two group.

**Fig.2:**
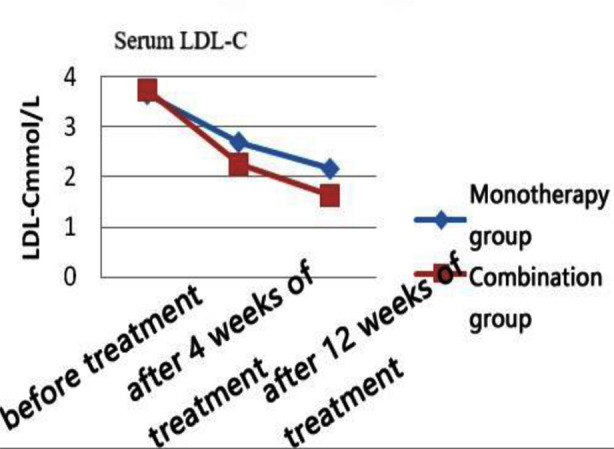
LDL-C levels at each time point in the two groups

**Fig.3:**
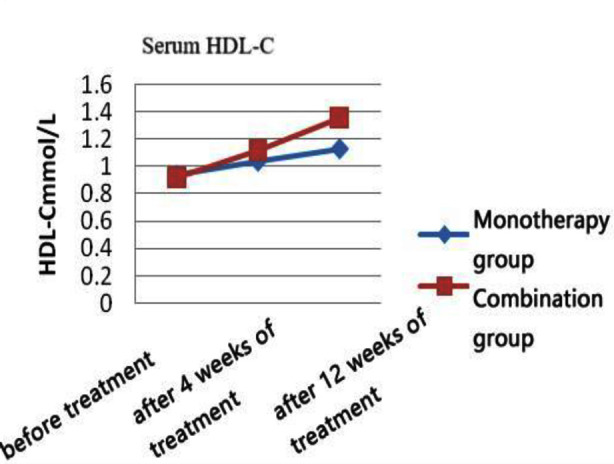
HDL -C levels at each time point in the two groups.

**Fig.4:**
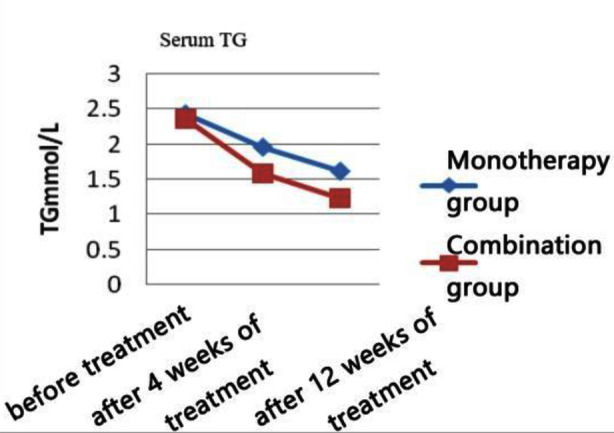
TG levels at each time point in the two groups.

There was no statistically significant difference between the two groups in terms of general information comparison (all p>0.05) (Table-I). Post-treatment, the levels of TC, LDL-C, and TG in both groups of patients were significantly reduced compared to pre-treatment (p<0.05). At four and 12 weeks of treatment, the levels of TC, LDL-C, and TG in the combination therapy group were more significantly reduced than those in the monotherapy group (p<0.05). Post-treatment, the HDL-C levels of both groups of patients increased compared to pre-treatment (p<0.05), and the combination therapy group showed a more significant increase than the monotherapy group at week 12 of treatment (p<0.05) (Table-II and Fig.1-4).

**Table-I:** Baseline characteristics of patients in the two groups.

	Monotherapy group (n=37)	Combination group (n=48)	P-values
Age (years)	67.3±7.1	68.7±5.5	0.32
Female [n (%)]	20(54.1%)	27(56.3)	0.84
Hypertension [n (%)]	22(59.5)	29(60.4)	0.77
Diabetes [n (%)]	6(16.2)	8(16.7)	0.45
Smoking [n (%)]	4(10.8)	5(10.4)	0.95
TC (mmol/L)	5.19±0.85	5.28±0.94	0.67
LDL-C (mmol/L)	3.65±0.63	3.74±1.01	0.60
HDL-C (mmol/L)	0.94±0.22	0.92±0.23	0.66
TG (mmol/L)	2.43±0.99	2.37±1.06	0.79

**Table-II:** blood lipids before and after treatment (*χ̅*±*S*, mmol/L)

	Monotherapy group(n=37)			Combination group (n=48)		
	Pre-treatment	Week 4	Week 12	Pre-treatment	Week 4	Week 12
TC	5.19±0.85	4.22±0.58^a^	3.86±0.62a	5.28±0.94	3.76±0.70^ab^	3.27±0.50^ab^
LDL-C	3.65±0.63	2.69±0.48^a^	2.16±0.38a	3.74±1.01	2.25±0.39^ab^	1.62±0.21^ab^
HDL-C	0.94±0.22	1.04±0.18^a^	1.13±0.21a	0.92±0.23	1.12±0.20^a^	1.36±0.16^ab^
TG	2.43±0.99	1.96±0.69^a^	1.62±0.48a	2.37±1.06	1.59±0.50^ab^	1.23±0.24^ab^

***Note:*** Repeated measurement analysis of variance and separate effect analysis of grouping factors were used.

a p<0.05 when compared with the same group before treatment, ^b^p<0.05 as compared between the two groups at the same treatment time.

Post-treatment, the levels of hs CRP and IL-6 in both groups of patients were significantly reduced compared to pre-treatment (p<0.05); at 4-12 weeks of treatment, the degree of decrease in IL-6 levels in the combination therapy group was higher than that in the monotherapy group during the same period (p<0.05); At 12 weeks of treatment, the degree of decrease in hsCRP in the combination therapy group was higher than that in the monotherapy group (p<0.05) (Table-III and IV).

**Table-III:** hsCRP before and after treatment (mg/L).

Treatment		hsCRP before and after treatment			Sum	F	P
		Pre-treatment	Week 4	Week 12			
Monotherapy n=37	*χ̅*	4.6	3.2	2.8	3.5	17.299	0.000
	S	1.9	1.4	1.6	1.8		
Combination n=48	*χ̅*	4.8	2.8	1.7	3.1	59.801	0.000
	S	1.7	1.4	0.86	1.9		
Sum n=85	*χ̅*	4.7	3.0	2.2	3.3	67.271*	0.000*
	S	1.8	1.4	1.4	1.8		
*t*		-0.35	1.42	4.1	4.888*	(F=4.322, p=0.000) #	
*p*		0.725	0.159	0.000	0.030*		

* F statistic and P values of main effect; ^#^F statistics and P values of interaction effects.

**Table-IV:** IL-6 before and after treatment (ng/L)

Treatment		IL-6 before and after treatment				Sum	F	P
		Pre-treatment	Week 4	Week 12				
Monotherapy n=37	*χ̅*	179.3	139.3	110.2		143.0	84.681	0.000
	S	27.3	27.5	12.9	36.8			
Combination n=48	*χ̅*	182.4	121.7	98.3		133.5	230.249	0.000
	S	27.9	18.3	11.0	41.5			
Sum n=85	*χ̅*	181.1	129.3	102.4		137.6	282.110*	0.000*
	S	27.5	24.2	13.7	39.7			
*t*		-0.514	3.552	5.342		11.232*	(F=5.562,p=0.005) #	
*p*		0.609	0.001	0.000	0.001*			

Both groups experienced serious adverse reactions during the treatment process; four abdominal distension (10.8%), one nausea (2.7%), and one vomiting (2.7%) were reported for the monotherapy group, and two abdominal distension (4.1%) and zero nausea and zero vomiting for the combination treatment group, there were no significant differences (p>0.05).

## DISCUSSION

The results of this study showed that the TC, LDL-C and TG were significantly improved in the two groups after four and 12 weeks of treatment. The reduction of LDL-C in the combination treatment group was significant than that of the monotherapy group. Both groups can increase HDL-C, and the combined treatment group has a more significant effect. Therefore, it was concluded that atorvastatin combined with Zhibitai is more effective to improve blood lipid components.^6^ Secondly, the results of the present study confirmed that the combination treatment reduced IL-6 and hs-CRP more significantly than the monotherapy, indicating that the combination treatment more effectively inhibited the chronic low-degree inflammatory response with a more marked effect on the atherosclerotic inflammatory response compared with the monotherapy.^7^,^8^ Thirdly, the safety profile of the combination treatment group was favorable.^9^ The incidence of gastrointestinal reactions such as abdominal distension, nausea, and diarrhea was even lower in the combination treatment group than those of the monotherapy group, and this was possibly contributed by the ingredients of Zhibitai capsules such as hawthorn, Rhizoma alismatis, and Atractylodes macrocephala, which promote the secretion of digestive juice and consequently reduce gastrointestinal reactions such as diarrhea and abdominal distension. The difference between the two groups, however, was not statistically significant. The results of this study are consistent with multiple research findings.^10^,^11^

The mechanism of atherosclerosis has been studied for more than ten decades. Studies worldwide showed that inflammation is the underlying mechanism triggering atherosclerosis.^12^,^13^ LDL and VLDL can be oxidized and modified to activate inflammatory reaction and promote the development and progression of atherosclerosis, while HDL acts as an anti-atherosclerotic agent by transporting antioxidant enzymes to terminate lipid oxidation.^14^ CRP is an acute reactive protein synthesized and secreted by the liver and a non-specific indicator for acute phase inflammation, while hs-CRP, which is useful in detecting CRP at low concentration and represents low-level inflammation, is considered as a marker for cardiovascular disease.^15^,^16^ IL-6 and hs CRP promote inflammatory response and involve in the development and progression of atherosclerosis.^17^ Although medium-dose statin treatment can reduce LDL-C, the effect on TG and HDL-C was relatively low.^18^ Although statins combined with fibrates can increase HDL-C, there are risks of rhabdomyolysis, renal damage, and increased blood homocysteine. Zhibitai capsule is a compound traditional Chinese medicine preparation with red rice, hawthorn, Rhizoma alismatis, and Atractylodes macrocephala as the main components.^19^ It has mild to moderate lipid-lowering effects with rare adverse reactions. Red rice contains many acid lovastatins, hawthorn can promote digestion, activate lipase and enhance fat catabolism.^20^

### Limitations:

It includes the relatively small number of enrolled patients, lack of evidence from large-scale multicenter trials, the relatively short follow-up period, and lack of follow-up observation of long-term efficacy and primary and secondary cardiovascular endpoints.

## CONCLUSION

Atorvastatin combined with Zhibitai capsules ensures in efficacy and safety and is significantly effective in the prevention and control of atherosclerosis.

### Authors’ Contributions:

**HZ** and **SK:** Designed this study, literature search and prepared this manuscript, and are responsible and accountable for the accuracy or integrity of the work.

**NX** and **LZ, LB** collected, and analyzed clinical data. Critical Review.

All authors have read and approved the final version of the manuscript.
